# The function of histone methyltransferase SETDB1 and its roles in liver cancer

**DOI:** 10.3389/fcell.2024.1500263

**Published:** 2024-11-08

**Authors:** Enxiang Zhang, Pingping He

**Affiliations:** State Key Laboratory for Macromolecule Drugs and Large-scale Manufacturing, School of Pharmaceutical Sciences and food engineering, Liaocheng University, Liaocheng, China

**Keywords:** liver cancer, SETDB1, DNA methylation, H3K9 trimethylation, tumor immunotherapy

## Abstract

Epigenetic alterations in gene expression have been implicated in cancer development and tumor immune escape, with posttranslational histone or non-histone modifications representing attractive targets for disease surveillance and therapy. SET domain bifurcated 1 (SETDB1) is a histone lysine methyltransferase that reversibly catalyzes the di- and tri-methylation of histone 3 lysine 9 (H3K9) on euchromatin, inhibiting gene transcription within these regions and facilitating the switch from euchromatic to heterochromatic states. Emerging evidence suggests that SETDB1 amplification and aberrant activation are significantly associated with poor prognosis in hepatocellular carcinoma (HCC), and contribute to HCC development, immune escape, and immune checkpoint blockade (ICB) resistance. Here, we provide an updated overview of the cellular and molecular effects of SETDB1 activity in hepatocarcinogenesis and progression and focus on studies linking its function to immunotherapy for HCC, and present current challenges and future perspectives for targeting SETDB1 in HCC treatment.

## 1 Introduction

Hepatocellular carcinoma (HCC) is one of the most common malignant tumors, and it is also one of the most common malignant tumors in Asian population. Its high morbidity and mortality pose a serious threat to human health. Despite recent advances in the diagnosis and treatment of HCC, its complex pathogenesis is still not fully understood. As an important field for the study of gene expression regulation, epigenetics has played a key role in the occurrence and development of many diseases (Ling and Rönn, 2019; Baccarelli and Ordovás, 2023; Hardy and Mann, 2016). Similar to other cancers, HCC is a heterogeneous disease driven by progressive genetic aberrations, including tumor suppressor gene silencing, oncogene activation, and chromosomal abnormalities (Hanahan and Weinberg, 2011).

SET domain bifurcated 1 (SETDB1) is an important histone 3 lysine 9 (H3K9) methyltransferase, which is found to be abnormally expressed in several types of cancers (Strepkos et al., 2021). Several studies have shown that SETDB1 amplification plays a key role in tumorigenesis and progression, such as promoting cell proliferation, migration, invasion, epithelial-mesenchymal transition (EMT), metastasis, drug resistance, and immune evasion (Orouji et al., 2019; Yu et al., 2020). However, the specific role and regulatory mechanism of SETDB1 in HCC have not been fully elucidated.

Several recent studies have focused on the role of SETDB1 in the development and progression of HCC. For example, one study found that the high expression of SETDB1 was closely related to the clinical stage and prognosis of HCC, suggesting that SETDB1 may be an important prognostic indicator of HCC (Wong et al., 2016). Another study found that SETDB1 could affect the biological behavior of liver cancer cells by regulating the methylation status of certain key genes (Fei et al., 2015a). Moreover, epigenetic dysregulation is one of the most important hallmarkers of tumorigenesis (Hanahan and Weinberg, 2011). Existing evidence has identified epigenetic alterations as the driver of immune escape (Gomez et al., 2020), so targeting epigenetic molecules such as SETDB1 may enhance the immune response and overcome immune checkpoint blockade (ICB) resistance in HCC cells.

In this review, we discuss in detail the role and molecular mechanisms of SETDB1 overexpression in HCC, particularly its role in regulating tumor immune responses. Finally, we discuss the current challenges and perspectives of targeting SETDB1 for HCC treatment and suggest some future research directions in the field of SETDB1 research.

## 2 Structure and cellular distribution of SETDB1

### 2.1 The structure of SETDB1

SETDB1, also known as ERG-associated protein with SET domain (ESET), is a member of the histone lysine N-terminal methyltransferase family. SETDB1 is expressed in human, mouse, monkey, sheep, and other mammals. Human SETDB1 is mapped to chromosome 1q21.3, consisting of 1,291 amino acids with a molecular weight of 143.1 kDa (Markouli et al., 2021a). Both the SET domain and the amino acid sequence are conserved during evolution. The domain composition of SETDB1 includes an N-terminal part containing a methyl-CpG-binding domain (MBD) and three Tudor domains, and a C-terminal part with a pre-SET, a SET, and a post-SET domain (Torrano et al., 2019).

Three Tudor domains anchor SETDB1 to arginine and lysine residues on histone or non-histone substrates and are critical to the formation of complexes for proteins that regulate transcriptional activity through chromatin modification, such as Histone Deacetylase 1/2 (HDAC1/2) and Kruppel-associated box-Zinc Finger Proteins-KRAB-Associated Protein-1 (KRAB-ZFP-KAP-1) (Schultz et al., 2002). In addition, the Tudor domains can also regulate snRNP processing in Cajal bodies (Terns and Terns, 2001).

MBD is functional, containing two DNA-interacting arginine residues that facilitate DNA binding and coupling the DNA methyl-CpG binding function to the H3K9 methylation function by interacting with DNA methyltransferase 3 (DNMT3) and inducing gene silencing (Ohki et al., 2001; Ho et al., 2008). Thus, the interaction between DNA methylation and histone methylation may promote epigenetic marking (Kang, 2015). Alternatively, the N-terminal part of SETDB1 contains two nuclear export signals (NES) and two nuclear localization signals (NLS), which regulate the localization of SETDB1 in cells (Cho et al., 2013).

The presence of the C-terminal pre-SET, SET, and post-SET domains of SETDB1 is critical for the activity of the protein methyltransferase (Yang et al., 2002). The bifurcated SET domain is the main region of catalytic activity and is separated by a large piece of insertion (Harte et al., 1999). It has been shown that ubiquitination at the SET insertion site K867 is essential for the integrity of mammalian SETDB1 enzymatic activity (Sun and Fang, 2016; Ishimoto et al., 2016). The presence of an evolutionarily conserved K867 in the insertion fragment, which can constitute monobititination in an E3-independent manner and is essential for maintaining the enzymatic integrity of SETDB1 (Sun and Fang, 2016). The SET domain recruits S-adenosyl methionine (SAM) as a cofactor to methylate the ε-amino group of lysine residues during catalysis (Wang et al., 2003). In addition, the SET domain is arranged in a helix structure, linked to the antiparallel double-stranded β-sheets by amino acid loops of different lengths that intercept the branched SET domains. This blocked amino acids chain, preserved by evolution, was shown to significantly regulate the activity of the SETDB1 protein (Wu et al., 2010).

**FIGURE 1:**
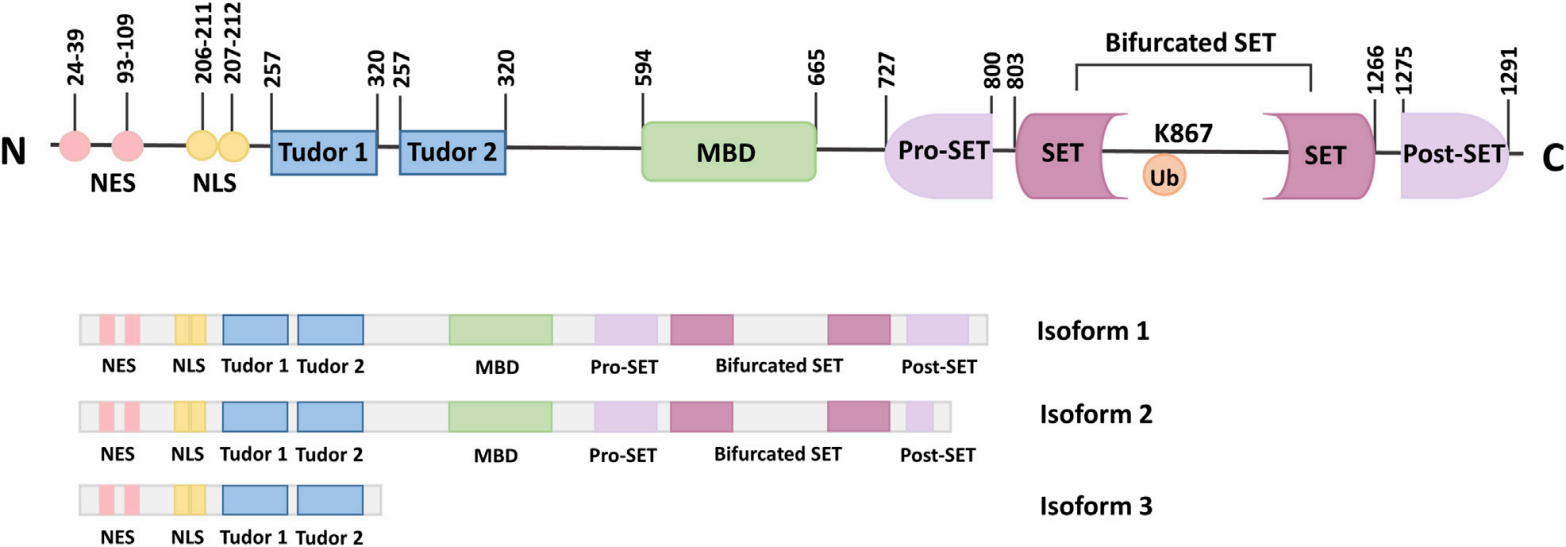
Schematic representation of the SETDB1 gene, functional domains and its isoforms. The N-terminal of SETDB1 contains two Tudor domains, a CpG DNA methyl-binding domain (MBD) and a branched SET domain, and the C-terminal contains a pre-SET, a SET, and a post-SET domain. SETDB1 has three different Isoforms, which are produced by alternative splicing.

Three isoforms of the SETDB1 gene have been identified, which result from alternative splicing. Isoforms one is encoded by the complete longest transcript code, containing all the necessary exons and domains, and is widely expressed (Markouli et al., 2021a; Blackburn et al., 2003). Although isoform 2 is the shorter splice variant, it still has all the important domains similar to isoform 1. In addition, isoform 2 also contains pre-SET, SET, and post-SET domains, as well as two Tudor domains and an MDB domain like isoform 1. In contrast to isoform 1, isoform 3 lacks the C-terminus and comprises only 400 amino acids at the N-terminus (Figure 1) (Markouli et al., 2021b; Karanth et al., 2017).

### 2.2 The distribution of SETDB1

SETDB1 is normally expressed in nuclei, but there are few studies on the subcellular localization of human SETDB1. Tachibana et al. found that SETDB1 was mainly expressed in the cytoplasm by establishing a human cell line expressing enhanced green fluorescent protein fused to hSETDB1 (Tachibana et al., 2015). This is because SETDB1 undergoes proteasomal degradation, resulting in its export to the cytoplasm (Tachibana et al., 2015). Nevertheless, SETDB1 is also widely distributed in the nucleus, especially in heterochromatin regions. It regulates gene expression by interacting with histones and other proteins to form a stable complex (Loyola et al., 2009). In addition, SETDB1 can also interact with other epigenetic marks, such as methylation and acetylation, to further affect gene expression.

## 3 Biological functions of SETDB1

**FIGURE 2:**
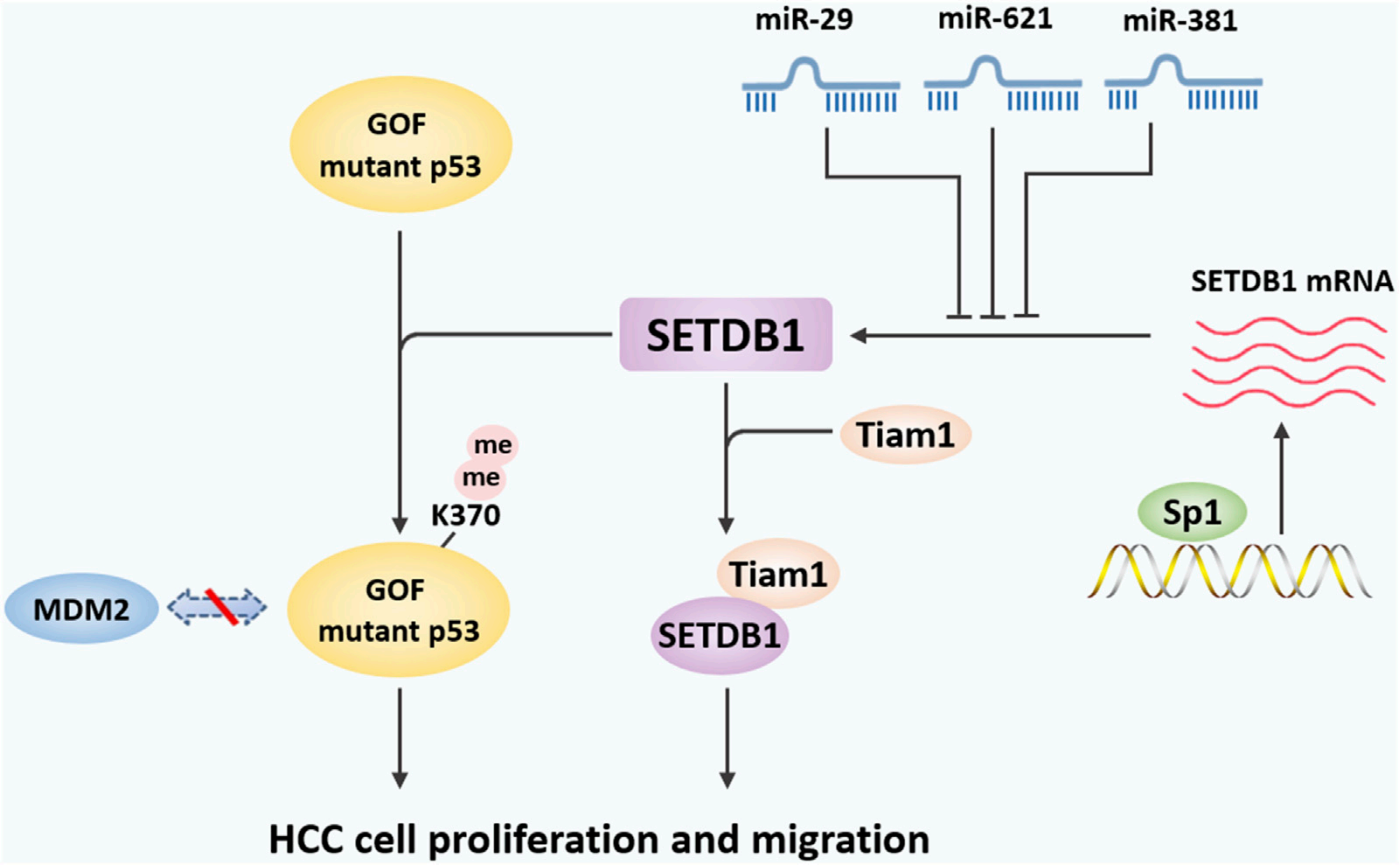
Biological functions of SETDB1. The SET domain of SETDB1 uses the cofactor S-adenosylmethionine (SAM) as the methyl donor and human ATFA-related regulator (hAM) as the inducer to trimethylate histone 3 lysine 9, thereby changing chromatin composition and inhibiting gene expression to participate in complex and diverse physiological processes. SETDB1 also maintains the structural integrity of PML-NBs and is essential for various biological processes mediated by PML-NBs. SETDB1 is involved in the suppression of ERV elements to promote genome stabilization and can regulate oocyte and spermatogenic meiosis. In addition, SETDB1 is also essential for maintaining X chromosome inactivation (Xi) and preventing inflammatory response. SETDB1 can promote cell division by interfering with the activity of p53 and AKT and interact with Cyclin D1 and c-MYC to induce cell proliferation. Finally, SETDB1 is involved in early embryonic development by regulating the expression of genes involved in pluripotency and trophectoderm development, and it can regulate the development of the nervous system by inhibiting differentiation markers.

The main role of SETDB1 is to participate in processes such as histone methylation, transcriptional repression and chromatin gene silencing, and chromatin remodeling in cells (Strepkos et al., 2021) (Figure 2). At the same time, as a genome-wide chromatin modifier, SETDB1 also has many other physiological functions, including regulating PML-NB(Promyelocytic leukaemia nuclear bodies) complex formation (Cho et al., 2013), mediating X chromosome inactivation (Minkovsky et al., 2014), inhibiting endogenous retrovirus (Matsui et al., 2010), regulating cell proliferation (Chen et al., 2017), regulating inflammatory response (Juznić et al., 2021), and regulating helper T cell differentiation (Hu et al., 2021). In addition, during normal development, SETDB1 also plays an important role in the development of the central nervous system (Lohmann et al., 2010). What’s more, SETDB1 is strongly associated with a variety of diseases, such as neuro-related diseases and a variety of tumors (Strepkos et al., 2021; Markouli et al., 2021b).

### 3.1 SETDB1 regulates epigenetic effects

Histone methylation is a major regulator of epigenetic modification and plays a key role in gene expression. Among them, histone lysine methylation is one of the important regulatory factors, which is related to the regulation of complex physiological activities and the malignant transformation of tumors (Li et al., 2011).

Existing studies have found that H3K9 is the main substrate for SETDB1. SETDB1 can specifically methylate H3K9 residues, thereby maintaining the structure of DNA and controlling gene expression by regulating the degree of DNA compression (Fischle et al., 2003). In this reaction, the SET domain of SETDB1 uses the cofactor S-adenosine methionine (SAM) as the methyl donor to methylate the ε-amino-methylation of the lysine residue (Herz et al., 2013). SETDB1 individually di-methylates H3K9. Human ATFa-associated modulator (hAM) can induce the conversion of H3 lysine di-methylation to trimethylation and promote the gene repression activity of SETDB1 through a SAM-dependent mechanism, which is achieved by binding to SETDB1 to form the SETDB1/hAM complex (Wang et al., 2003). The interaction of SETDB1 with hAM, while not a prerequisite for its function, can enhance its activity (Wang et al., 2003). In addition, H3K9 trimethylation is associated with gene suppression, while H3K9 monomethylation is associated with gene excitation (Barski et al., 2007). Thus, binding to hAM increases SETDB1-dependent transcriptional inhibition on chromatin templates.

### 3.2 SETDB1 regulates PML-NBs formation

Promyelocytic leukaemia nuclear bodies (PML-NBs) are a ribosome containing a variety of proteins, which play an important role in many biological processes, including gene transcription, tumor suppression, apoptosis, neovascularization, DNA repair, antiviral response, and maintenance of genome stability (Bernardi and Pandolfi, 2007; de Thé et al., 2012). SETDB1 has been established as an integral component of the PML-NB structure. Cho et al. demonstrated the binding of endogenous SETDB1 and PML proteins at the stage of mouse lytic development and demonstrated their colocalization on PML-NBs (Cho et al., 2011). In this complex, SETDB1 has a dual function. On the one hand, SETDB1 is essential for maintaining the structural integrity of PML-NBs, which is mediated by the physical interaction of SETDB1 with PML protein through its SIM motif (Kang, 2015; Cho et al., 2011). Knockdown of SETDB1 results in the decomposition of PML-NBs, whereas degradation of PML by arsenic treatment results in the disappearance of SETDB1 foci (Cho et al., 2011). On the other hand, SETDB1 is a transcriptional regulator of PML-NBs related genes. Chromatin immunoprecipitation (CHIP) assay revealed that SETDB1 binds the promoter of DNA binding inhibitor 2 (ID2) within the PML-NBs framework and inhibits its expression by installing H3K9 methylation (Cho et al., 2011).

### 3.3 SETDB1 mediates X-Chromosome inactivation

X-Chromosome inactivation (XCI) is an epigenetic silence caused by heterochromatin formation early in the development of female mammalian embryos that persists throughout the life cycle of somatic cells (Minkovsky et al., 2014). SETDB1 has also been found to be associated with XCI, which promotes gene silencing and maintains XCI by altering the conformation of the entire inactivated X chromosome (Keniry et al., 2016). During XCI, activating transcription factor 7-interacting protein (ATF7IP or MCAF1) interacts with methyl-CpG-binding domain protein 1 (MBD1) to favor SETDB1-mediated H3K9me3 and hence heterochromatin formation, leading to H3K9 trimethylation on the X chromosome inactivation (Xi) and random silencing of one of the two X chromosomes in female cells (Minkovsky et al., 2014). Further studies confirmed that the formation of MBD1-chromatin assembly factor-1 (CAF-1) chaperone complex initiates the formation of the transcriptional repressive complex by mediating the recruitment of SETDB1 to the CAF-1 large subunit and maintaining XCI in somatic cells (Reese et al., 2003; Sarraf and Stancheva, 2004; Ichimura et al., 2005). The heterochromatin structure is inherited during DNA replication through association with MBD1 and ATF7IP (Sarraf and Stancheva, 2004). In addition, ATF7IP can also mediate the location of SETDB1 in the nucleus and increase the ubiquitination of SETDB1, thereby enhancing its enzymatic activity (Tsusaka et al., 2019).

### 3.4 SETDB1 mediated silencing of retroelements

Endogenous retroviruses (ERVs) are a subclass of viral retroelements that contain long terminal repeats scattered in euchromatin regions of mammalian DNA and account for approximately 8% of the human genome (Maksakova et al., 2006; Geis and Goff, 2020). Although retrotransposition contributes to genome diversification evolution and adaptation, it can also lead to genome instability, insertional mutations, or transcriptional perturbations (Mager and Stoye, 2015). Thus, integrated retroelements are usually transcriptionally silenced by DNA methylation and/or H3K9 trimethylation. The study found that SETDB1 inhibited the expression of ERVs, thereby minimizing the likelihood that they would alter DNA. Tan et al. demonstrated that the reconstruction of ERV expression in SETDB1 knockout mice upregulated the expression of other neighboring genes, a large proportion of which produced chimeric transcripts with ERVs or possessed ERVs within 10 kb of their starting site (Tan et al., 2012). Specifically, ERVs silencing is initiated by the recruitment of KRAB-associated protein-1 (KAP1) to the target by members of the KRAB zinc finger protein (KRAB-ZFP) family (Fukuda and Shinkai, 2020). KAP1 contains a RING-B box-coiled helix domain, a heterochromatin protein 1(HP1) binding domain, and a PHD-bromodomain for recruiting SETDB1 to gene promoters, thereby establishing a H3K9me3-silenced chromatin state on KAP1-targeted genes (Fukuda and Shinkai, 2020).

Mouse embryonic stem cells (mESCs) have been found to enhance SETDB1 recruitment to ERV retrotranspose and form KAP1 inhibitory complexes to repress proviral molecules (Thompson et al., 2015). Moreover, RNA binding protein and transcription cofactor heterogeneous nuclear ribonucleoprotein K (hnRNP K), which are required for SETDB1-dependent proviral silencing processes, act as a binding partner of the SETDB1-KAP1 complex through direct interaction in mESCs (Thompson et al., 2015). Notably, Fukuda et al. found that retroelement silencing factor 1 (RESF1) knockout mouse embryonic stem cells reduced SETDB1 enrichment at the provirus and ERV sites (Fukuda et al., 2018). This interaction suggests that RESF1 may also play a role in SETDB1-mediated ERV inhibition by modulating the action of SETDB1. Recently, studies of SETDB1 knockout in adult mice and differentiated cells have shown that SETDB1 also inhibits reverse transcriptional elements in somatic cells, suggesting that SETDB1 has a persistent role in inhibiting ERVs expression even after early developmental stages (Kato et al., 2018).

In addition, SETDB1-dependent ERV inhibition has also been shown to be associated with the regulation of CD4^+^ T cell differentiation and the evasion recognition by the immune system of cancer cells. Takikita et al. demonstrated that SETDB1 is required for CD4^+^ T cells to acquire and maintain T helper 2 (Th2) responses (Takikita et al., 2016). And Th2-differentiated, SETDB1 knockdown CD4^+^ T cells were unable to maintain their Th2 differentiation when exposed to Th1-inducing signals (Takikita et al., 2016). On the other hand, SETDB1 inhibits ERV expression through H3K9 methylation to prevent ERV-induced B cell immune response, thereby enabling acute myeloid leukemia (AML) cells to evade innate immunity (Cuellar et al., 2017).

### 3.5 SETDB1 regulates cell division and proliferation

SETDB1 has been shown to interact with cell cycle regulators to exert cell cycle effects. SETDB1 enhances c-MYC and Cyclin D1 expression by promoting internal ribosome entry site (IRES)-mediated translation of MYC/CCND1 mRNA, leading to significant signaling of c-MYC, thereby promoting cell cycle progression and providing cells with a growth/self-renewal advantage (Xiao et al., 2018). P53 is a known central regulator of cell cycle progression and apoptosis, and many studies have shown that SETDB1 can inhibit p53 through methylation, thereby promoting cell proliferation (Strepkos et al., 2021). In addition, the regulation of SETDB1 on cell proliferation and survival is also related to the regulation of protein kinase B (protein kinase B, AKT) activity (Guo et al., 2019). Studies have shown that AKT undergoes SETDB1-mediated lysine methylation to promote its activation. Mechanistically, the interaction of phosphatidylinositol (3,4,5)-triphosphate with AKT promotes its interaction with SETDB1, which in turn promotes AKT methylation and thus maintains AKT phosphorylation (Guo et al., 2019).

### 3.6 SETDB1 regulates the inflammatory response

SETDB1 also inhibits Toll-like receptor 4 (TLR4)-mediated inflammatory responses in macrophages (Hachiya et al., 2016). Mechanistically, SETDB1 deficiency reduced basal H3K9 methylation and increased TLR4-mediated recruitment of nuclear factor κB (NF-κB) to the proximal promoter region of interleukin-6 (IL-6), thereby accelerating IL-6 promoter activity. In addition, upon lipopolysaccharide (LPS) stimulation, H3K9 methylation was reduced, which was associated with the recruitment of NF-κB p65 to this site, thereby activating IL-6 transcription (Hachiya et al., 2016). This suggests that SETDB1-mediated resting-state H3K9 methylation may act as a gatekeeper in regulating the inflammatory response, contributing to the balance between suppression and activation of proinflammatory cytokines.

In addition, SETDB1 is also necessary for intestinal epithelial differentiation and prevention of intestinal inflammation. Deletion of SETDB1 results in de-silencing of ERVs, DNA damage and inflammation, ultimately leading to intestinal epithelial cell death (Juznić et al., 2021). The study found that while mucosal SETDB1 expression was not impaired in the vast majority of inflammatory bowel disease (IBD) patients, a comparison of IBD and non-IBD exome revealed overexpression of rare missense variants of SETDB1 in IBD individuals, some of which are predicted to be associated with loss of function and may be associated with the pathogenesis of intestinal inflammation (Juznić et al., 2021).

### 3.7 SETDB1 is involved in early embryo development

Studies have shown that the SETDB1 gene is actively transcribed during the blastocyst stage of embryogenesis and controls the expression of genes associated with pluripotency and trophoectodermal development (Cho et al., 2013; Lohmann et al., 2010; Bilodeau et al., 2009). In contrast to other H3K9-specific histone lysine methyltransferases, SETDB1 dysfunction was found to induce the earliest lethality around peri-implantation (Peters et al., 2001; Tachibana et al., 2002; Tachibana et al., 2005). After knockout of SETDB1 on embryonic stem cells (ES), significant downregulation of the pluripotency controlling transcription factors Oct4, Nanog, and Sox2 was observed, as well as upregulation of differentiation markers (Cdx2, Gata2, and Hand1) (Dong et al., 2023). Bilodeau et al. also found that SETDB1 knockdown induced upregulation of several differentiation markers, including paired-box 3 (Pax3), homeobox a1 (Hoxa1), and nuclear receptor subfamily 2 group F member 2 (Nr2f2), suggesting that ES cells would transfer to differentiation in the absence of SETDB1 (Blackburn et al., 2003). In addition, the catalytic activity of SETDB1 is required in meiosis and early oogenesis, and the absence of SETDB1 leads to a reduction in the number of mature eggs (Kim et al., 2016; Eymery et al., 2016).

### 3.8 SETDB1 coordinates the central nervous system development

Additionally, SETDB1 has also been shown to be involved in the early development of the central nervous system. A study by Tan et al. showed that SETDB1 is highly expressed in the early stages of mouse brain development, where it binds to the promoters of astroglia-related genes (GFAP, SOX9), suppressing their transcription through H3K9me3 and preventing premature formation of astrocytes (Tan et al., 2012). In addition, SETDB1 expression decreased with the increase of embryonic age. SETDB1 ablation leads to early lethality and severe defects in brain development, with enhanced astrocyte formation and inhibition of neurogenesis, which reveals the importance of SETDB1 in brain development (Tan et al., 2012). Fei et al. found that SETDB1 can interact with polycomb repressive complex 2 (PRC2) to repress genes involved in neuronal differentiation (Fei et al., 2015b). This finding suggests that SETDB1 can use an alternative mechanism to repress gene regulation that is distinct from its direct enzymatic activity.

## 4 Upstream regulation of SETDB1

The expression and activity of SETDB1 are regulated by several aspects. In 2006, Ryu et al. identified specific protein 1 (Sp1) and specific protein 3 (Sp3) as transcriptional activators of SETDB1, which can bind to the promoter of SETDB1 to activate transcription of the gene (Ryu et al., 2006). Miramycin, a clinically approved DNA-binding antitumor antibiotic rich in guanosine-cytosine, can interfere with the DNA binding of these Sp family transcription factors (Osada et al., 2013). It has been reported that miramycin inhibits the basal activity of the SETDB1 gene promoter in a dose-dependent manner. In addition, the combination of minomycin and hemiamine downregulated the expression of SETDB1 and decreased the hypermethylation of H3K9 (Ryu et al., 2006). Moreover, another transcription factor 4 (TCF4) can also directly bind to the promoter of SETDB1 to enhance its expression (Shang et al., 2021). As mentioned above, SETDB1 promotes cell cycle progression by increasing c-MYC expression. Indeed, increased c-MYC can also positively feedback by directly binding to the SETDB1 promoter to regulate SETDB1 expression and enhance its transcription (Xiao et al., 2018).

MicroRNAs (miRNAs) are a class of single-stranded non-coding RNAs with an intrinsic length of approximately 21–25 nt (Lewis et al., 2005), which can also negatively regulate SETDB1 expression by targeting the 3′-UTR of its mRNA (Shao et al., 2019). Shao et al. found that miR-621 expression was low in HCC tissues and cells, and this low expression was associated with poor prognosis in HCC patients. Further studies showed that miR-621 and miR-29 directly bound to the 3′-UTR of SETDB1 to suppress its expression (Shao et al., 2019). In addition, miR-409–3p was found to negatively regulate SETDB1 expression in non-small cell lung cancer (NSCLC) (Liu et al., 2020).

Ubiquitination of SETDB1 is critical for its stability, methyltransferase activity, nuclear localization, and function. As mentioned above, ATF7IP inhibits SETDB1 nuclear export by binding to its N-terminal region and increases its ubiquitination, thus enhancing its methyltransferase activity, while ATF7IP deficiency promotes the proteasomal degradation of SETDB1, thereby attenuating its expression (Tsusaka et al., 2019). Some studies have shown that disruption of the ATF7IP-SETDB1 complex in tumor cells can restore the expression of tumor antigens, which provides a rationale for cancer immunotherapy targeting SETDB1 or ATF7IP (Hu et al., 2021).

## 5 Role of SETDB1 in HCC

**FIGURE 3:**
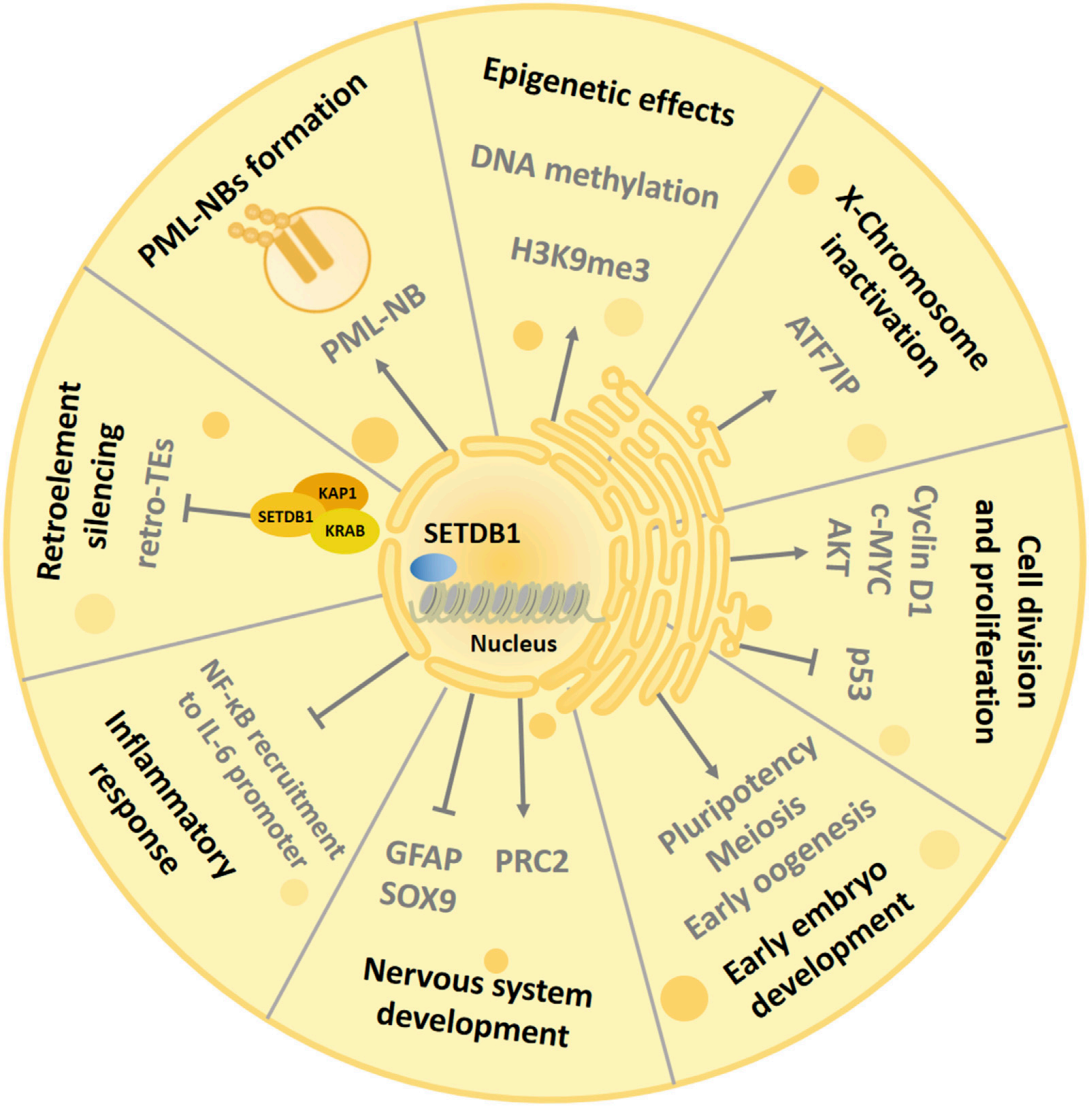
The mechanisms underlying the role of SETDB1 in HCC. In liver cancer, SETDB1 dimethylates gain-of-function (GOF) mutant P53 at K370 to prevent its degradation by MDM2-mediated ubiquitination, and then promotes liver cancer cell growth. SETDB1 also can cooperate with Tiam1 to promote the proliferation and migration of HCC cells by forming the SETDB1-Tiam1 complex. In addition, miR-29, miR-621, and miR-381 directly target the 3′-UTR of SETDB1 and inhibits its expression, while overexpression of specific protein 1 (Sp1) transcriptively enhanced SETDB1 expression.

Recent studies have shown that there is a close relationship between cancer formation and epigenetic dysregulation (Vendetti and Rudin, 2013). Moreover, epigenetic mechanisms often cooperate with genetic mechanisms in the process of malignant tumor development caused by changes in chromatin status (Hanahan and Weinberg, 2011). It has been reported that the histone methyltransferase SETDB1 is overexpressed in most cancer types, and its induced aberrant methylation of H3K9 is an important player in epigenetics and is involved in the genesis of a variety of cancers. According to TCGA data, SETDB1 is amplified in 10.8% of liver cancers, 9.1% of breast cancers, 8.4% of bladder cancers, 7.4% of ovarian cancers, and 6% of uterine cancers, and mutated in about 5% of melanomas. Importantly, SETDB1 can suppress tumor innate immunogenicity and evade immune responses by inhibiting transposable elements enriched genomic regions (Griffin et al., 2021). The following is the detailed summary of the role of SETDB1 in HCC and the mechanisms involved (Figure 3).

### 5.1 Amplification and overexpression of SETDB1 in HCC

Epigenetic dysregulation plays an important role in the development of liver cancer. In 2015, Wong et al. used transcriptome sequencing to examine the expression of 591 epigenetic regulators in hepatitis B-related human HCC (Wong et al., 2016). The results suggest that aberrant expression of epigenetic regulators is a common event in HCC. Importantly, SETDB1 was the most significantly upregulated epigenetic regulator in human HCC. SETDB1 overexpression is significantly correlated with HCC progression, tumor aggressiveness (such as tumor microsatellite formation and metastasis), and poor prognosis of HCC patients. Further studies found that SETDB1 knockdown reduced the proliferative and migratory capacity of HCC cells, suppressed orthotopic tumorigenicity, and abolished the formation of lung metastasis. Mechanistically, the frequent upregulation of SETDB1 in human HCC is attributed to a recurrent copy gain of SETDB1 on chromosome 1q21. In addition, overexpression of specific protein 1 (Sp1) transcriptively enhanced SETDB1 expression. The above results indicate that multiple mechanisms of SETDB1 activation at the chromosomal, transcriptional, and post-transcriptional levels jointly contribute to SETDB1 upregulation in human HCC.

In another study, Fei et al. examined SETDB1 expression in six HCC samples adjacent to normal tissue and obtained similar results (Fei et al., 2015a). SETDB1 expression was elevated in 4 of 6 pairs of tumor tissues compared with control tissues. Furthermore, SETDB1 is overexpressed with modest copy number gain in HCC, and the hotspot gain-of-function (GOF) TP53 mutations including R249 S are associated with this overexpression (Fei et al., 2015a). In HCC cell lines carrying the R249 S mutation, inhibition of SETDB1 by siRNA or shRNA suppressed cell proliferation. Taken together, SETDB1 overexpression is mediated by several complementary mechanisms of action, suggesting that upregulation of SETDB1 may be a marker of HCC progression.

### 5.2 SETDB1 aggravates HCC by methylating p53

SETDB1-mediated dimethylation of the tumor suppressor p53 plays an important role in the progression and metastasis of HCC. TP53 encodes the human tumor suppressor p53 and is one of the most frequently mutated genes in HCC. TP53 is reported to be mutated in about 50% of human tumors, including HCC (Brosh and Rotter, 2009; Shen and Ong, 1996; Mutational hotspot in the p53 gene in human hepatocellular carcinomas Hsu IC et al., 1991). The investigators found that the proportion of TP53 mutations was significantly increased in HCC tumor samples with SETDB1 copy number gain or overexpression. Moreover, in HCC cell lines bearing R249 S mutation, SETDB1 inactivation inhibited cell growth. Immunoprecipitation assay showed that SETDB1 can form a complex with p53, especially mutant p53. To further determine the methylation modification of p53 by SETDB1, Fei et al. used p53 peptide and synthetic K370me1 as substrates and SAM as the methyl donor of SETDB1, respectively, and showed that SETDB1 may be a p53 methylase that mainly converts K370me1 to K370me2 (Fei et al., 2015a).

Further studies revealed the regulatory mechanism of SETDB1 on p53 protein stability. It has been previously shown that p53 methylation affects p53 protein stability (Chuikov et al., 2004). The researchers introduced wild-type or mutant p53 into HCT116 p53-null cells and treated them with the protein synthesis inhibitor cycloheximide. As expected, wild-type p53 disappeared rapidly, whereas R249 S mutant p53 showed greater stability. When SETDB1 is knocked down, p53R249 S turned over at an accelerated rate. In addition, SETDB1 knockdown increased the ubiquitination level of p53 and increased the association of MDM2 with p53. Furthermore, SETDB1 inhibition also reduced the S15 phosphorylation of p53, which has been shown to stabilize p53 by preventing p53 ubiquitination (Meek, 2009). Finally, in HCCLM3 xenotransplantation models in nude mice, SETDB1 knockdown significantly inhibited tumor growth and increased cell differentiation. Collectively, SETDB1 overexpressed in HCC formed a complex with p53 and catalyzed p53K370 dimethylation. Attenuation of SETDB1 reduced the levels of p53K370me2 and subsequently resulted in increased recognition and degradation of p53 by MDM2 (Meek, 2009).

### 5.3 SETDB1 promotes cells proliferation and migration by interacting with Tiam1 in HCC

HCC has a high recurrence rate due to its poor prognosis and high rates of intrahepatic and extrahepatic metastasis (Budhu et al., 2006). However, the metastatic potential of HCC cells is affected by a variety of factors, including cell intrinsic characteristics and external microenvironmental factors (Fidler, 2003). Previous studies have shown that HCC metastasis is caused by the T-lymphom invasion and metastasis gene (Tiam1) gene, a member of the Dbl gene family that controls guanine nucleotide exchange factors (GEFs) (Huang et al., 2013). A study by Zhang et al. showed that SETDB1 is also closely related to Tiam1-induced HCC metastasis (Zhang et al., 2018). The data showed that the average expression levels of SETDB1 and Tiam1 in HCC samples were significantly higher than those in normal tissues. Importantly, the expression of SETDB1 was positively correlated with Tiam1. Further, the direct interaction between SETDB1 and Tiam1 was further determined by glutathione-S-transferase (GST) pull-down and cross-linked immunoprecipitation (CLIP) tests. Specifically, SETDB1 cooperates with Tiam1 to promote the proliferation and migration of HCC cells by forming the SETDB1-Tiam1 complex. In addition, overexpression of SETDB1 promoted the proliferation, migration and EMT of HCC cells, while the effects were reversed after Tiam1 knockdown (Zhang et al., 2018).

### 5.4 Regulation of SETDB1 by microRNAs in HCC

As mentioned above, miR-29 has been reported to be downregulated frequently in human HCC (Wong et al., 2014). Later studies found that in the TCGA sample set, miR-29 family members were significantly downregulated and negatively correlated with SETDB1 expression levels (Wong et al., 2016). Wong et al. further found that overexpression of miR-29 significantly inhibited luciferase activity in wild-type SETDB1 3′-UTR labeled reporter cells. However, this effect was attenuated in miR-29 binding site mutants, thus confirming the specific negative regulatory function of miR-29 on SETDB1 (Wong et al., 2016).

In addition to miR-29, miR-621 has been shown to reduce SETDB1 expression by directly targeting the 3′-UTR of SETDB1 and enhancing the radiosensitivity of HCC cells (Shao et al., 2019). MiR-621 is located on chromosome 13q, and the absence of miR-621 can upregulate the cell cycle regulation gene, leading to the proliferation of liver cancer cells. Moreover, miR-621 is one of the miRNAs with the most significant expression difference between HCC and paracancer tissues (Shao et al., 2019). Shao et al. further used the dual luciferase reporter gene system to prove that SETDB1 is the direct target gene of miR-621, and that miR-621 can activate p53 signaling pathway by inhibiting SETDB1, and ultimately enhance the radiosensitivity of HCC cells (Shao et al., 2019). A recent study showed that SETDB1 also seems to have a potential binding with miR-381, and enhancer of zeste homolog 2 (EZH2) inhibits the expression of miR-381 by promoting H3K27me3 activity in its promoter region, which can promote the expression of SETDB1 (Zhou et al., 2022). In conclusion, the results of several studies suggest that miRNAs may be negative regulators of SETDB1 and that the loss of specific miRNAs in human HCC may contribute to the upregulation of SETDB1 by removing its post-transcriptional control. More in-depth research is needed to reveal other miRNAs that can regulate SETDB1.

### 5.5 SETDB1 suppressed HBV replication in HCC by Sirt2.5 inhibition

Hepatitis B virus (HBV) infection is one of main factors for HCC progression, so targeting HBV replication is ideal method for HCC repression and clinical therapeutical application development.

The human sirtuin 2 functioned as a deacetylase, which could promote HCC progression by enhancing HBV replication. Sirt2 owns 5 different isoforms as Sirt2.1, Sirt 2.2, Sirt 2.3, Sirt 2.4, Sirt 2.5. Different from other isoform, Sirt2.5 was in nuclei, because the loss of nuclei export sequence. Upon HBV infection all isoforms were enhanced but Sirt2.5 did not influence HBV replication via Akt but due to epigenetic regulation. As established, Sirt2.5 was in nuclei and its binding with Akt was weakened upon HBV infection. In Sirt2.5 overexpression HBV-infected cells, some HKMTs including SETDB1 were recruited to bind with Sirt 2.5, following inhibition of cccDNA transcription (Piracha et al., 2024). So, targeting SETDB1/Sirt2.5 axis could be a great target for HCC due to HBV infection.

## 6 Therapeutic targeting of SETDB1 in HCC

### 6.1 Targeting SETDB1-mediated AKT methylation in HCC

In addition to P53, AKT has also been reported to be a substrate for SETDB1 methylation, involved in tumorigenesis and leading to cancer cell growth and increased glycolysis (Guo et al., 2019; Li et al., 2006). As part of the inositol triphosphate (IP3)/AKT pathway, AKT is an important regulator of cell proliferation and survival (Manning and Toker, 2017). Studies have shown that SETDB1 trimethylates the K140 and K142 sites of AKT to promote its phosphorylation on T308 and S473 sites and activation, which is antagonized by lysine demethylase 4B (KDM4B). Moreover, in NSCLC, SETDB1 activates K63-linked AKT ubiquitination by trimethylating AKT at K64 and subsequently by recruiting Jumonji domain protein 2A (JMJD2A) and E3 ligase TRAF6 to the AKT complex (Piracha et al., 2024). Similarly, in colorectal cancer, overexpression of SETDB1 promotes cell proliferation by activating AKT, while inhibition of SETDB1 enhances cetuximab sensitivity in colorectal cancer therapy (Hou et al., 2020).

In liver cancer, activation of the AKT pathway has been shown to be an important risk factor for early recurrence and poor prognosis in patients (Buontempo et al., 2011). Moreover, the PI3K/AKT/mTOR signaling pathway proteins were significantly elevated in PET/CT-positive HCC patients, suggesting that the activation of this pathway may be a key factor in the glycolytic phenotype of HCC cells (An et al., 2022). Several mTOR inhibitors have been tested to treat HCC but have failed in clinical trials, so targeting SETDB1-mediated AKT methylation is a promising strategy for HCC.

### 6.2 Targeting interference to immune system of SETDB1 in HCC

Immunotherapy has shown considerable efficacy in several cancer treatments. Preclinical and clinical studies have shown that ICB therapy provides survival benefits to more patients with liver cancer. A combination of anti the programmed cell death protein 1/programmed cell death ligand 1 (PD-1/PD-L1) and anti-cytotoxic T-lymphocyte-associated protein 4 (CTLA-4) antibodies is currently being evaluated in clinical trials for liver cancer. Recent studies have shown that the expansion of SETDB1 in human tumors plays a crucial role in allowing tumor immune escape and resisting ICB (Lin et al., 2021).

By screening for chromatin regulators with CRISPR-Cas9, Griffin et al. identified that SETDB1 and other members of the HUSH and KAP1 complexes act as mediators of immune escape in ICB-treated mouse tumor models. When SETDB1 is targeted by CRISPR-Cas9 sgRNA, sensitivity to ICB therapy increases (Griffin et al., 2021). Further studies found that SETDB1 primarily inhibits a wide range of domains in the open genomic region that are rich in immune clusters and transposable elements (TE) associated with fragment replication events. SETDB1 loss suppresses potential TE-derived regulatory elements, immunostimulatory genes, and TE-encoded retrovirus antigens in these regions and triggers a TE-specific cytotoxic T cell response (Griffin et al., 2021).

In addition, the expression level of SETDB1 also affects the efficacy of ICB therapy. It has been reported that SETDB1-TRIM28 inhibition combined with PD-L1 elevation promotes micronucleus formation in the cytoplasm, thereby activating the cGAS (cyclic GMP-AMP synthase)-STING innate immune response pathway, and further increases the infiltration of CD8 T cells (Lin et al., 2021). The absence of SETDB1 can significantly improve the efficacy of anti-PD-L1 therapy. Therefore, it may be considered to combine SETDB1 inhibition with ICB in the treatment of HCC.

### 6.3 Targeting microRNAs to inhibit SETDB1 in HCC

As mentioned above, several miRNAs that can negatively regulate SETDB1 have been identified in HCC, including miR-29, miR-621, and miR-381, so targeting miRNAs is a viable approach to inhibit SETDB1. In addition, SETDB1 has been found to interact with the DNA methyltransferase DNMT3A. Interestingly, members of the miR-29 family have also been shown to target DNMT3 in HCC cells (Kogure et al., 2014). Low levels of miR-29 and DNMT3A modulation were associated with HCC invasiveness, while forced expression of miR-29 abrogated transforming growth factor (TGF)-β-induced E-cadherin supression (Kogure et al., 2014). Moreover, miR-29 levels are controlled to maintain the differentiated hepatocyte phenotype (Cicchini et al., 2015). Therefore, targeting miR-29 may affect HCC progression and metastasis by regulating the methylation activity of histone and DNA. In addition, after transfection of miR-621 in HCC cells, not only decreased SETDB1 expression, but also decreased cell survival, increased apoptosis, and increased DNA damage response index γ-H2AX were observed (Shao et al., 2019). Therefore, targeting miR-621 is a way to improve the sensitivity of HCC radiation therapy.

### 6.4 Application of SETDB1 inhibitors to reduce its function in HCC

Given the important role of SETDB1 in tumorigenesis, progression, metastasis, and tumor immune escape, the development of SETDB1 inhibitors is a promising strategy for cancer chemotherapy and immunotherapy. However, unfortunately, SETDB1 inhibitors used in preclinical trials are mostly nonselective compounds. This is due to the lack of a SET domain crystal structure, which is challenged by its bifurcated characteristic. At present, there are few studies on the use of SETDB1 inhibitors to inhibit HCC progression, so we summarized SETDB1 inhibitors for other cancer therapies.

Miramycin A and miramycin analogue EC-8042 have been reported to inhibit SETDB1 expression in melanoma to reduce the tumor growth (Federico et al., 2020). Cardamosin can inhibit breast tumor growth by inhibiting SETDB1 and prevents the enrichment of breast cancer stem-like cells when combined with chemotherapy drugs (Jia et al., 2016). Piperlongumine, a natural alkaloid compound, has also been found to reduce SETDB1 expression to induce PARP cleavage and FOSB expression in breast cancer cell lines, leading to cell death (Park et al., 2019). BIX-01294 (CAS 935693–62–2), a G9a HMTase inhibitor, also reduced SETDB1 expression levels in melanoma cell lines. The combination of BIX-01294 with BRAF and MEK inhibitors for the treatment of BRAF-mutated cells showed a high level of synergistic effects (Orouji et al., 2019). In addition, SAM hydrolase inhibitor DZNep increased the anti-proliferative and pro-apoptotic activities of lung cancer cells by down-regulating SETDB1 (Lee and Kim, 2013). Arsenic trioxide (As_2_O_3_) has been reported to severely reduce SETDB1 levels by inducing promyelocytic leukemia protein degradation (Cho et al., 2013) (R, R)-59 is a selective SETDB1-TTD small molecule inhibitor screened based on this concept that has been shown to inhibit SETDB1 in acute monocytic leukemia cells (Guo et al., 2021). Of interest, several chemotherapeutic agents, including paclitaxel (PTX), cisplatin, doxorubicin, and 5-fluorouracil, have also been shown to inhibit SETDB1 expression at both transcriptional and protein levels (Na et al., 2016). Among them, PTX inhibited SETDB1 expression in a p53-dependent manner, while cisplatin and doxorubicin reduced H3K9me3 levels and inhibited tumor growth (Na et al., 2016; Na and Kim, 2018).

## 7 Prospects and future research directions

**FIGURE 4:**
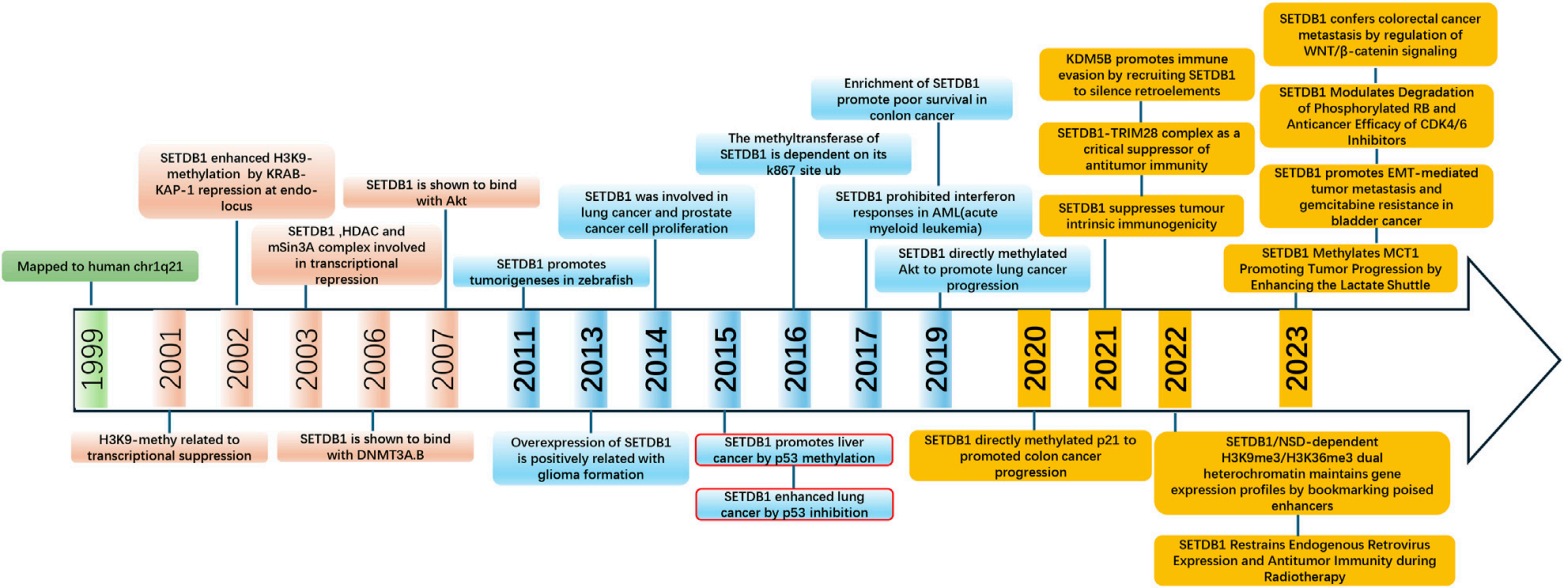
Timeline of major discoveries in SETDB1 studies, including basic science and clinical translational studies.

There is no doubt that alterations in epigenetic modifications and chromatin remodeling are involved in the pathogenesis of many diseases, including cancer (Figure 4). As a key H3K9 methyltransferase, SETDB1 is a promising candidate. So far, the abnormal expression and poor prognosis of SETDB1 in various cancers have demonstrated its role as an oncogene. Also, great progress has been made in elucidating the underlying mechanisms of SETDB1 regulation and its functional implication in different cancers. However, the specific role of SETDB1 in HCC and its regulation of a series of interaction signaling pathways have not been fully elucidated. Based on the review of the relevant studies of SETDB1 in HCC, we believe that future research directions are as follows:

Firstly, existing studies have shown that SETDB1 is the most significantly upregulated epigenetic regulator in human HCC, and its overexpression affects HCC progression, tumor invasiveness and poor prognosis. However, the difference of SETDB1 in different pathological stages of liver cancer has not been deeply studied. It has been reported that the high expression of SETDB1 is associated with the advanced pathological status of patients with NSCLC (Inoue et al., 2015), but other studies have found that SETDB1 is strongly expressed in the early stage of NSCLC (Wu et al., 2014; Lafuente-Sanchis et al., 2016). Interestingly, high levels of SETDB1 mRNA were found to persist in all stages of cancer in NSCLC patients, suggesting that SETDB1 has different roles at different stages of tumorigenesis (Cruz-Tapias et al., 2019). Therefore, relevant studies are needed to clarify the differences of SETDB1 in different stages of HCC, to provide the possibility of using SETDB1 as a biomarker for early diagnosis of HCC and a potential therapeutic target.

Second, recent studies on SETDB1 have highlighted its important role in regulating cancer through methylation of non-histone proteins, which further expands the downstream mechanism of SETDB1. However, whether SETDB1 can methylate other non-histone proteins in HCC and what its mechanism still needs to be further screened and explored. There is also an urgent need to clarify the specificity of SETDB1 binding to a specific substrate (histone or non-histone) in HCC.

At present, SETDB1 inhibition has been shown to significantly improve the sensitivity of liver cancer radiation therapy, so combining SETDB1 inhibition with the currently used liver cancer treatment methods, such as chemotherapy, radiotherapy or gene therapy, may have a good therapeutic effect. In addition, transposable factors and immune clusters have been found to be silenced by SETDB1-dependent H3K9 methylation in cancer, suggesting that SETDB1 is a negative regulator of tumor intrinsic immunity. Therefore, SETDB1 can be used as a candidate target for immunotherapy and be considered for use in combination with ICB inhibitors for HCC therapy. On the other hand, it also suggests that the development of SETDB1 inhibitors with high specificity, low toxicity and high efficiency may provide a new option for SETDB1 targeted liver cancer treatment. Most of the recently reported effective SETDB1 antagonists are non-specific, and since most are cytotoxic chemotherapeutic agents, off-target effects and side effects are inevitable. The potential idea for developing selective SETDB1 inhibitors is to specifically block the SETDB1-TTD interaction or compete with SAM to inhibit SETDB1 activity. In addition, active substances such as paclitaxel have been found to inhibit the expression of SETDB1, and many natural active substances have hepatoprotective effects, so it is possible to consider screening SETDB1 inhibitors among them.

In addition, SETDB1 is not the only enzyme that mediates H3K9 methylation; other methyltransferases can also mediate this histone labeling. Therefore, the complex interactions between SETDB1 and other epigenetic enzymes, such as other methylases or acetylases, should also be considered when inhibiting SETDB1 to minimize off-target side effects of its therapeutic targeting.

Moreover, therapeutic RNAs targeting SETDB1, including small interfering RNAs (siRNAs), antisense oligonucleotides (ASOs), or large RNAs such as mRNAs, long non-coding RNAs (lncRNAs), and cyclic RNAs, also represent an alternative strategy for developing disease therapies. For example, overexpression of Hox antisense intergenic RNA (HOTAIR), a functional non-protein long non-coding RNA (lncRNA), has been shown to promote cancer progression by different mechanisms. Studies have shown that HOTAIR-mediated direct inhibition of miR-7 in breast cancer stem cells can upregulate SETDB1 and inhibit E-cadherin, thus favoring EMT (Zhang et al., 2014). HOTAIR-mediated regulation of SETDB1 in HCC has not been reported, so future studies can focus on its role in HCC.

Immunotherapy has shown considerable efficacy in several cancer treatments. Preclinical and clinical studies have shown that ICB therapy provides survival benefits to more patients with liver cancer. A combination of anti the programmed cell death protein 1/programmed cell death ligand 1 (PD-1/PD-L1) and anti-cytotoxic T-lymphocyte-associated protein 4 (CTLA-4) antibodies is currently being evaluated in clinical trials for liver cancer. But SETDB1 overexpression exerted resistant sometimes. So, targeting SETDB1 and combination with ICB could be an ideal therapy for HCC.

But due to the lack of structure of whole SETDB1,there was no direct inhibitors developed currently,so the specification of its structure and agonist development will be most promising for HCC or other diseases resulting from SETDB1 abnormality.

Finally, further studies, including animal model experiments, organoid model experiments and clinical trials, are needed to confirm whether SETDB1 is feasible as a therapeutic target and suitable for human clinical treatment. We hope that this review effectively presents the extensive research to better understand the function of SETDB1 in liver cancer. We believe that future studies will make greater progress and provide new strategies for the early diagnosis and effective treatment of liver cancer patients.
